# Aerial low-frequency hearing in captive and free-ranging harbour seals (*Phoca vitulina*) measured using auditory brainstem responses

**DOI:** 10.1007/s00359-016-1126-8

**Published:** 2016-10-31

**Authors:** Klaus Lucke, Gordon D. Hastie, Kerstin Ternes, Bernie McConnell, Simon Moss, Deborah J. F. Russell, Heike Weber, Vincent M. Janik

**Affiliations:** 1 Centre for Marine Science and Technology, Curtin University, Perth, Australia; 2 Wageningen Marine Research, Wageningen University and Research, Wageningen, The Netherlands; 3 Sea Mammal Research Unit, Scottish Oceans Institute, University of St Andrews, St Andrews, UK; 4 Zoo Duisburg, Duisburg, Germany; 5 Tierpark Nordhorn, Nordhorn, Germany

**Keywords:** Harbour seal, *Phoca vitulina*, Hearing, ABR, Low frequency

## Abstract

The hearing sensitivity of 18 free-ranging and 10 captive harbour seals (*Phoca vitulina*) to aerial sounds was measured in the presence of typical environmental noise through auditory brainstem response measurements. A focus was put on the comparative hearing sensitivity at low frequencies. Low- and mid-frequency thresholds appeared to be elevated in both captive and free-ranging seals, but this is likely due to masking effects and limitations of the methodology used. The data also showed individual variability in hearing sensitivity with probable age-related hearing loss found in two old harbour seals. These results suggest that the acoustic sensitivity of free-ranging animals was not negatively affected by the soundscape they experienced in the wild.

## Introduction

Marine mammals have long been recognised as hearing specialists and perhaps the most vulnerable group of all aquatic animals to noise pollution at sea (National Research Council 2003; Tyack 2008; Reichmuth et al. 2013; Finneran 2015). The physical characteristics of the marine environment create a very different sensory landscape from terrestrial habitats. While light does not penetrate water as far as in air, acoustic information is transmitted much faster and with less attenuation in underwater environments. Furthermore, moving objects leave long-lasting hydrodynamic trails that can be used to trace them (Dehnhardt et al. 2001). Thus, marine organisms tend to employ different sensory modalities than terrestrial species in orientation, foraging, and communication. Some marine mammals, primarily the pinnipeds, lead a truly amphibious lifestyle, alternating between marine and terrestrial habitats. To effectively forage and communicate within these environments, their sensory systems have to be adapted to both media and the associated different sensory challenges and opportunities.

Noise can have a range of effects on animals, including hearing loss, increased stress, cognitive and developmental impairment, behavioural disruption, deterioration of body condition, and the induction of heart and other disease (Knight and Swaddle 2011; McGregor et al. 2013). For a single, localised and short-lived noise source, this may not be much of a concern. However, with the expansion in worldwide marine traffic and offshore industrial developments, sound is now being introduced to marine environments on a global scale. One of the most industrialised marine environments in the world is the North Sea. Anthropogenic activities here include shipping, the use of seismic air guns for oil and gas exploration and the construction of oil and gas platforms as well as offshore wind farms. This area is also used by harbour seals (*Phoca vitulina*, Linnaeus), and the overlap between their at-sea distribution and sound producing activities has led to concerns about the potential impacts of sound on this species (Hastie et al. 2015). With sound being an efficient vector for transporting information in both media, harbour seals show a strong dependence on the production and perception of sounds both in air and underwater (Wartzok and Ketten 1999), especially in courtship behaviour and breeding interactions (Hanggi and Schusterman 1994; Burns 2002, Van Parijs and Kovacs 2002; Van Parijs et al. 2000, 2003; Hayes et al. 2004) as well as mother–pup interactions (Renouf 1984; Perry and Renouf 1988). To date, hearing studies on captive animals have shown that harbour seals have an acute sense of hearing in air and underwater (Bullock et al. 1971; Terhune 1991; Kastak and Schusterman 1998; Wolski et al. 2003; Reichmuth et al. 2013) with functional hearing ranging from at least 100 Hz up to 33 kHz in air and 51 kHz underwater (Reichmuth et al. 2013; see Cunningham and Reichmuth 2016 for high-frequency sensitivity). Despite this, there is a relative paucity of data on the hearing sensitivities of wild pinnipeds. To address this, we obtained auditory measurements on seals living in the North Sea during brief capture–release sessions in The Wash, UK, and compared them with measurements that we took from captive animals that lived in a comparatively low-noise environment.

Two methods are available to measure hearing thresholds in harbour seals—the classical psychophysical method (Møhl 1968) which provides the most accurate threshold information and more recently an electrophysiological approach (Wolski et al. 2003). Due to the relatively long-time investment required for psychophysical auditory studies for training and data acquisition, only a limited number of harbour seals of different sex and age classes have been measured with this method (Møhl 1968; Bullock et al. 1971; Terhune 1989, 1991; Kastak and Schusterman 1998; Terhune and Turnbull 1995; Wolski et al. 2003; Kastelein et al. 2009a, b; Reichmuth et al. 2013). The second method uses auditory brainstem responses (ABRs) which can be measured from the skin surface when a subject receives an acoustic stimulus (Burkard et al. 2007). The neuronal responses generated within the first 10 ms are likely to originate from the acoustic nerve and the auditory brainstem. These early ABRs allow an efficient and fast measurement of hearing thresholds and have been used successfully in studies on harbour seals (Bullock et al. 1971; Wolski et al. 2003) as well as other pinniped species (Houser et al. 2007; Mulsow and Reichmuth 2010; Ruser et al. 2014). In all of these studies, the thresholds of the individuals tested were relatively consistent at high frequencies, but showed marked individual differences in the low frequencies. Our study focused on these low frequencies, since the main energy of noise pollution in the North Sea is below 2 kHz (OSPAR Commission 2009) and any noise induced hearing impairment would most likely occur in or near this frequency range. While it was a key aspect of this study to investigate low-frequency sensitivity in wild seals, the short-term nature of our access to these animals was a limiting factor and dictated our choice of ABRs as a method. The strength of the ABR method is that it allows quick measurements of hearing sensitivity in animals which are otherwise not accessible for such tests. However, its use at low frequencies is not well established. Thus, our study aimed at using ABRs at low frequencies to assess their usefulness for further studies of hearing thresholds in the bandwidth that most noise pollution occurs in.

## Methods

The auditory sensitivity of 18 harbour seals of varying age and sex (see Table 1) was tested on sandbanks in The Wash, on the east coast of the U.K., in January 2012. In February 2013, the auditory sensitivity of ten additional harbour seals, also of varying age and sex (see Table 1), was tested at the Zoo Duisburg and Tierpark Nordhorn in Germany. These animals had been either kept in these facilities for all of their adult lives or were born there.

**Table 1:** Location and date of auditory measurements and information on subjects tested

Test environment	Location	Date	Subject	Sex	Age class	Age (years)^a^
Laboratory	Zoo Duisburg, GER	16.02.2013	Db01	Female	Adult	25
		16.02.2013	Db02	Male	Adult	28
		16.02.2013	Db03	Female	Subadult	1
		16.02.2013	Db04	Female	Adult	40
		16.02.2013	Db05	Male	Adult	25
	Tierpark Nordhorn, GER	18.02.2013	Nh01	Female	Adult	17
		18.02.2013	Nh02	Male	Subadult	1.5
		18.02.2013	Nh03	Male	Adult	33
		18.02.2013	Nh04	Female	Adult	27
		18.02.2013	Nh05	Male	Subadult	0.5
Free-ranging	The Wash, UK	22.01.2012	73279	Male	Juvenile	^b^
		22.01.2012	73280	Male	Juvenile	2
		23.01.2012	73282	Female	Adult	8
		23.01.2012	73283	Male	Adult	6.5
		23.01.2012	73286	Female	Adult	^b^
		23.01.2012	73287	Female	Adult	11.5
		24.01.2012	73288	Male	Adult	10.5
		24.01.2012	73290	Male	Adult	17
		24.01.2012	73291	Male	Adult	12
		24.01.2012	73292	Male	Adult	23
		24.01.2012	73293	Male	Adult	^b^
		24.01.2012	73294	Male	Adult	8.5
		25.01.2012	73296	Female	Adult	10.5
		25.01.2012	73297	Male	Adult	7
		25.01.2012	73295	Female	Adult	20.5
		25.01.2012	73298	Male	Adult	6
		25.01.2012	73299	Female	Adult	6.5
		25.01.2012	73300	Male	Adult	4

^a^At time of testing

^b^Age not determined

In The Wash, all animals were caught using hoop or seine nets. All procedures in the wild were carried out under Home Office Animals (Scientific Procedures) Act licence number 60/4009. Data on animal sex and weight were collected on site where ABR measurements were also taken. When possible, a tooth was extracted for aging purposes. Seals were aged by counting the growth layer groups in the cementum of an incisor tooth, using the method of Dietz et al. (1991). In the zoos, animals were moved to a veterinary lab for ABR measurements. Age was taken from zoo records.

Animals were given a premedication intramuscular injection of midazolam (Hypnovel^®^, females 0.09–0.13 mg/kg, males 0.09–0.38 mg/kg), after ~10 min they were anaesthetised with an injection of ketamine (Ketaset^®^, females 1.47–3.25 mg/kg, males 1.09–3.16 mg/kg) into the epidural sinus with a three and one half-inch spinal needle which was subsequently maintained in place. Additional intravenous doses of Ketaset^®^ were administered to maintain the desired anaesthesia, and additional intravenous doses of midazolam were administered to control muscular tremors, a side effect of ketamine anaesthesia.

### Acoustic stimulation

In-air hearing sensitivity was measured in sedated animals by measuring their ABRs. The hearing sensitivity was tested at 1.4, 2.0, and 2.8 kHz in all animals. Acoustic stimuli were presented binaurally via headphones (DT 48 A.0, Beyerdynamic GmbH and Co. KG) to the animals in trials of 512–2048 stimulus repetitions with a 5-dB step size in descending order. Short tone pips consisting of five cycles of cosine-gated sine waves were used as stimuli, with a duration between 5 ms (at 1 kHz) and 1.8 ms (at 2.8 kHz). Additional frequencies were tested (at half octave steps between 4 and 22.4 kHz) if time allowed or no reproducible results could be achieved at the initial test frequencies. At a repetition rate of 33.3 stimuli per second, the polarity of successive stimuli was inverted to avoid stimulus artefacts.

The signals emitted through the headphones were calibrated before the measurements using a frequency generator (Agilent, USA, type 33220A) for signal generation, an artificial ear (Brüel & Kjær, Denmark, type 4157) connected to a calibrated microphone (Brüel & Kjær, type 2669) and a conditioning amplifier (Brüel & Kjær, type NEXUS 2690) as receiver. The signals were calibrated in terms of sound pressure level (SPL rms) over the duration of the tone pips. All signals were visually inspected for spectral quality (distortion) on a digital oscilloscope (PeakTech Prüf- und Messtechnik GmbH, Germany, type 1205) over the tested frequency range. No signal distortion was documented for any of the frequencies and levels used in our study.

The starting sound pressure level for the first animal tested was chosen to be approximately 30 dB above the hearing threshold of harbour seals based on previous publications. In subsequent measurements on the remaining animals, levels were adjusted to start 30 dB above levels determined in the first animal. In addition, background electrical noise was measured in the absence of stimuli. In all trials (with and without acoustic stimulation), the headphones were held in place over the animal’s ear openings by one of the researchers. The opening of the outer ear canals was regularly checked and acoustic stimuli were only played when the outer ear canal was visibly open.

**Fig. 1:**
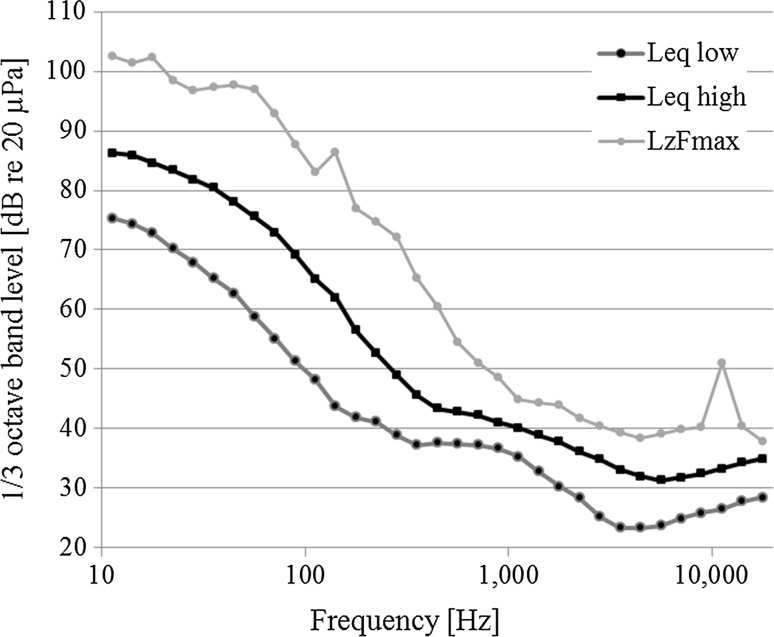
Equivalent continuous sound levels (*L*_eq_) at North Sea haul-out sites measured outside of the headphones. The figure shows measurements in high-noise and low-noise conditions. The *L*_*z*_ *F*_*max*_ *curve* shows the maximum values at the high-noise location during the 5-min measurement of the *L*_eq_ values (fast response time). All measurements were unweighted from 10 Hz to 20 kHz

Background noise was measured as the equivalent continuous sound pressure level (*L*_Zeq_, unweighted) using a Casella CEL-6X0 handheld sound level meter (Casella CEL Inc., Buffalo, NY, USA) (over a 5-min period in the frequency band of 6 Hz to 20 kHz (Fig. 1). In the laboratory settings, background noise was found to be 54 dB re 20 µPa (±2 dB). In the field, background noise was measured post hoc in the same weather conditions as when the initial auditory measurements were taken. On the sandbank, noise was mainly caused by wind and waves with an overall *L*_Zeq_ between 72 and 96 dB re 20 µPa (±2 dB) (1/3 octave band levels given in Fig. 1). In all experimental settings, the headphones provided 12-dB attenuation of the ambient noise (according to Beyerdynamic, technical specifications of DT 48.0A).

### Stimulus generation and response acquisition

The hardware setup for measuring the ABRs differed between the laboratory and field setting. On the sandbanks, stimulus generation, transmissions and recording of the neuronal responses were conducted using the custom made EVREST system (Finneran 2009) which includes a data acquisition board (NI PCI-6251, National Instruments, Austin, TX, USA). The acoustic stimuli were digitally generated, converted to analog at a 1-MHz update rate and 16-bit resolution, low-pass filtered at 250 kHz (Krohn-Hite Corporation, Brockton, MA, USA) and attenuated (over a range of 0–70 dB) before being presented to the animals via headphones. Recorded neuronal responses (digitisation rate 20 kHz) were amplified (94 dB) and bandpass filtered between 300 Hz and 3 kHz.

The audiometric measurements at the two facilities in Germany were conducted using a Tucker-Davis Technologies Workstation System 3 [Tucker-Davis Technologies (TDT), Alachua, FL, USA]. The acoustic stimuli were generated using the TDT software SigGen at a digitisation rate of 50 kHz. The recorded electrode responses were amplified (TDT RA4L; 20 dB gain), passed through an anti-aliasing filter, and led to an A/D converter (TDT RA16). Subsequently, the response (digitisation rate 25 kHz) was digitally filtered (high pass 300 Hz, low pass 3 kHz), written to a memory buffer and tested for the presence of signal artefacts. We used the TDT software BioSig to average the resulting potentials to allow an assessment of artefacts that indicates successful reception of the signal.

In all animals, the neuronal signals were measured with subdermal needle electrodes (NIHON-Kohden, Tokyo, Japan; 30 gauge) which were placed along the dorsal midline of the head: the active electrode on the vertex, 2 cm in front of the line between both ear openings, the ground electrode in the nape of the neck (i.e., 10–15 cm behind the ear-line, depending on the animal’s size) and the reference electrode another 10–15 cm further back. The input impedance between the electrodes was 1 kΩ or below during all measurements.

### Analysis

Neuronal waveforms were measured over a period of 10 ms after acoustic stimulation and averaged over the total number of presentations. The peaks of the recorded neuronal waveforms are numbered (I–VII) according to their succession (nomenclature of neuronal waves following Jewett and Williston 1971), with wave V being the most prominent wave which can also be identified more reliably at decreasing stimulus amplitude (under ideal conditions down to levels close to the hearing threshold). The amplitude of wave V of the response evoked by the tone pips was measured and used for threshold determination in this study.

In contrast to the EVREST system, the ‘TDT system 3’ provides no option for determining the threshold level based on the last positive identification of a neuronal response and the first miss. To allow for a comparative analysis of both data sets and reduce the influence of varying physiological noise levels between subjects and animal groups, a regression analysis of the wave V peak amplitudes was conducted after visual inspection of all recorded ABRs. A stimulus was considered as not perceived by the animal if an ABR was not detectable above the neuronal background noise level at each given frequency. Distorted measurements (due to technical reasons, strong movements of the animals, etc.) were not included into the regression analysis.

### Comparison of threshold levels

The hearing thresholds measured by Wolski et al. (2003) represent the only other auditory data achieved for a harbour seal with the same methodology (ABR). Expressing thresholds in terms of the energy content of the entire stimulus over time (SEL) as done by Wolski et al. (2003) is, strictly speaking, not appropriate as ABRs are an onset response. To allow for comparison, the data reported by Wolski et al. (2003) were converted into SPL levels. This allows for direct comparison with the thresholds reported in this study as well as behavioural hearing thresholds measured by Reichmuth et al. (2013) (for our data see Table 2).

**Table 2:** Average aerial hearing sensitivity of captive (laboratory) and free-ranging harbour seals in this study determined by measuring the auditory brainstem response as a function of frequency

Frequency (kHz)	Average laboratory			Average free-ranging		
	SPL (dB re 20 µPa)	*n*	s.d.	SPL (dB re 20 µPa)	*n*	s.d.
1.4	97	3	6.8	88	13	11.2
2	101	5	8.0	85	18	7.3
2.8	93	6	9.3	81	18	11.0
4				78	5	6.7
5.6				66	2	
8				54	2	
11.2				57	1	
16				34	2	
22.4				39	1	

Threshold values are given in terms of pressure (SPL) together with the number of animals tested (*n*) and the standard deviation (s.d.) of threshold values

## Results

**Fig. 2:**
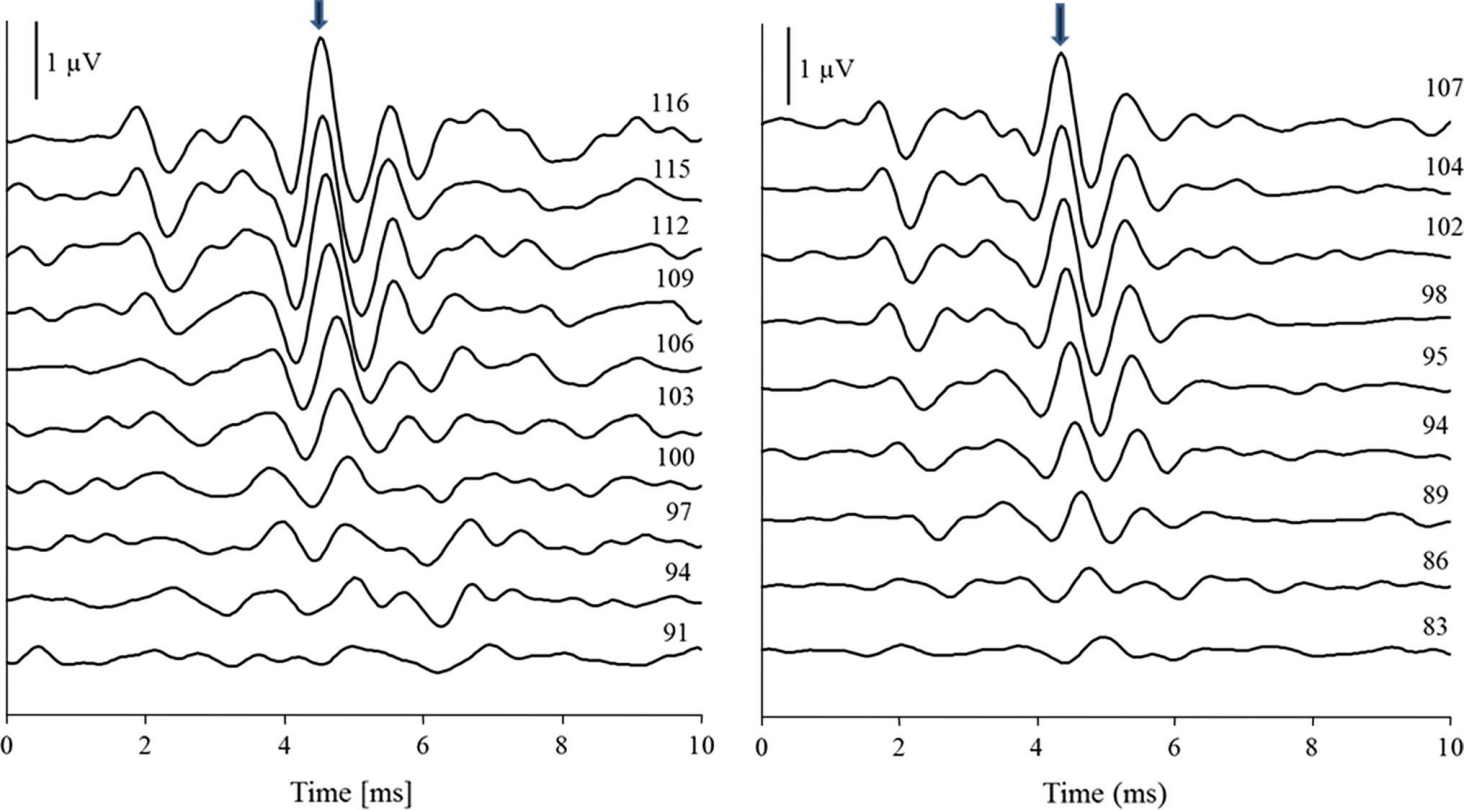
Tone-pip evoked potentials measured in an immobilised harbour seal, while animal was lying on a sandbank. Neuronal waveforms were measured over a period of 10 ms after acoustic stimulation and averaged over 512 presentations (epochs) at various levels (received level next to each ABR waveform in dB re 20 µPa). Signals were presented at 1.4 (*left*) and 2.8 kHz (*right*) across a range of amplitudes; recorded neuronal responses were filtered between 300 Hz and 2 kHz

**Fig. 3:**
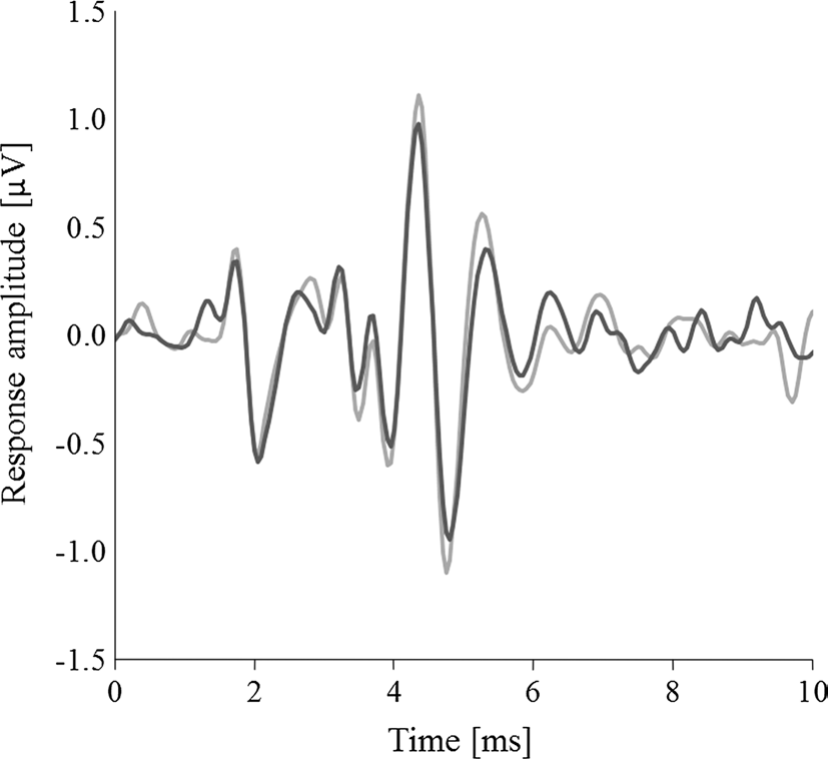
Overlaid waveforms representing two averages of 256 sweeps showing the variability in the ABR waves. Both sweeps were measured in the same animal at the same frequency (2.8 kHz) and stimulus level (105 dB re 20 µPa)

**Fig. 4:**
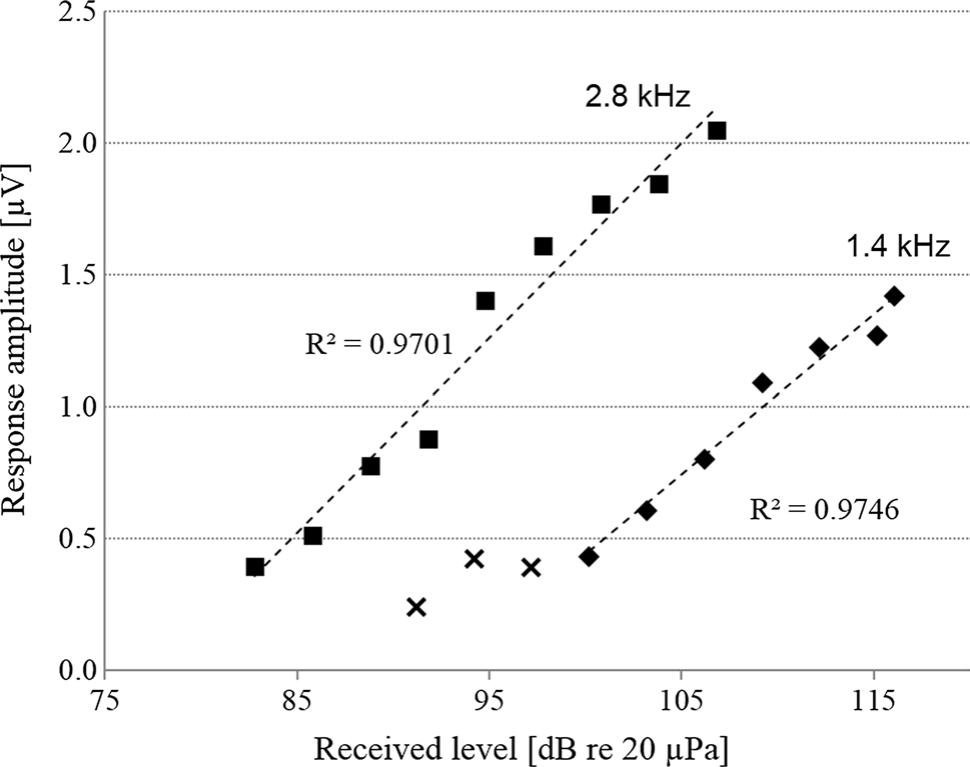
Results of the regression analysis of the wave V peak amplitudes measured in a free-ranging seal at 1.4 and 2.8 kHz. The *closed symbols* represent values included into the analysis, the *crosses* those values excluded from the regression analysis as ABRs were not detectable above the neuronal background noise at these received levels. The coefficient of determination (*r*-squared value) is given for both regression lines (*dashed lines*)

Hearing thresholds were measured for the target frequencies of 1.4, 2.0, and 2.8 kHz in all (18) free-ranging seals in The Wash in 2012 and in six of the ten animals tested in the zoos in 2013. Examples of the resulting neuronal waveforms measured after stimulation at 1.4 and 2.8 kHz are shown in Figs. 2 and 3. The amplitudes of the individual neuronal waves decreased at both frequencies with decreasing received sound pressure level while the latency of the waves increased. In comparison, the amplitudes recorded during stimulation with 1.4-kHz tone pips were lower than those elicited by 2.8 kHz tones (note the different range of SPL values in Fig. 2). Moreover, the latency of the neuronal waves differed between both frequencies, with the maximum positive peak of wave V (indicated by arrows in Fig. 2) appearing 4.35 ms after stimulus onset at the highest level measured at 2.8 kHz as compared to 4.55 ms at 1.4 kHz. Examples of the regression analysis conducted over the resulting wave V peak amplitudes at two frequencies are shown in Fig. 4.

**Fig. 5:**
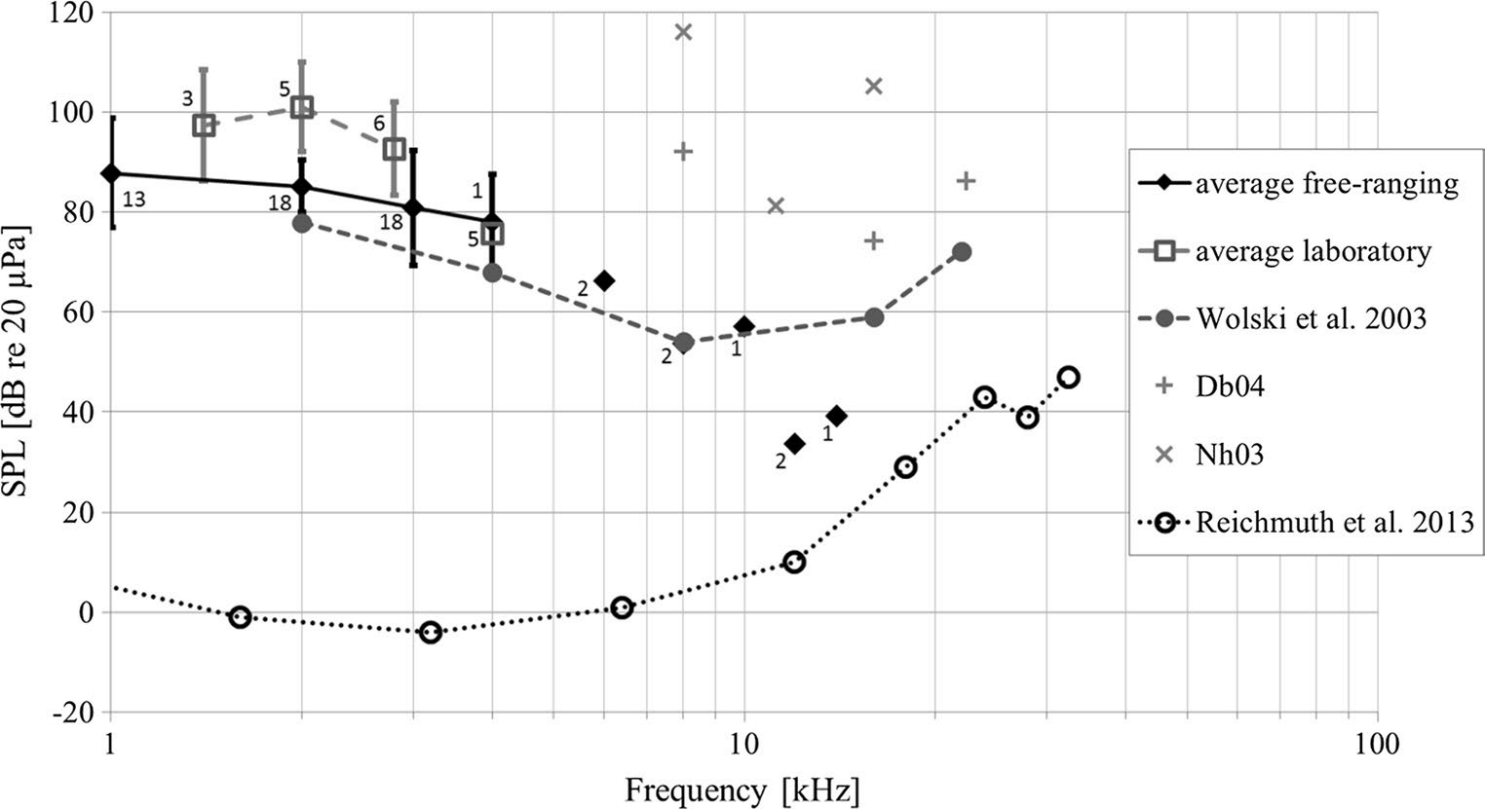
Average sound exposure levels of aerial hearing sensitivity of free-ranging (*diamonds*) and captive (*squares*) harbour seals as a function of frequency measured using the auditory brainstem response (ABR) method. *Numbers next to the symbols* give the number or animals analysed at a given frequency; *error bars* indicate the standard deviation of thresholds. For all our measurements, data from less than three animals are not connected to the hearing threshold line for that data set. *Grey crosses* and ‘*x*’ show the hearing thresholds achieved for two old animals, respectively (Db04, Nh03). Sound exposure levels of ABR hearing thresholds obtained in a captive harbour seal by Wolski et al. (2003)—achieved in a sound isolation box—are shown for comparison (*filled circles*). In addition, sound exposure levels of psychophysical hearing thresholds measured in a harbour seal in an unmasked acoustic environment (*open circles*; Reichmuth et al. 2013) are shown

In a few animals, additional frequencies covering the frequency range up to 22.4 kHz were measured. For details on sample sizes, see Table 2 and Fig. 5. In two animals, ABR responses allowed hearing thresholds to be determined at a single test frequency only (2 and 4 kHz, respectively), while in two older animals, only tests at the upper end of the frequency band tested (8–22.4 kHz) provided responses. In all but the two oldest harbour seals tested under laboratory conditions, the ABR patterns followed the typical mammalian pattern; auditory sensitivity increased with increasing frequency. Peak sensitivity was found at 16 kHz and tended to decrease toward higher frequencies (Fig. 5). Hearing thresholds in the free-ranging seals ranged at the low frequencies (≤4 kHz) from 53 to 103 dB re 20 μPa. In captive animals, the hearing thresholds ranged from 85 to 110 dB re 20 μPa. At frequencies above 4 kHz, the lowest threshold (34 dB re 20 μPa) was found at 16 kHz in a free-ranging seal. In the two older captive seals (33 and 40 years), no low-frequency hearing thresholds could be obtained, while at high frequencies some residual, but markedly reduced hearing sensitivity (ranging from 74 to 116 dB re 20 μPa as compared with 34 to 85 dB re 20 μPa in free-ranging seals) was measured.

The maximum differences in hearing sensitivity at low frequencies between the 18 free-ranging animals ranged from 26 dB (at 2 kHz, s.d. 7.3 dB) to 41 dB (at 2.8 kHz, s.d. 11 dB), while the captive animals showed a maximum individual difference in hearing sensitivity between 13 dB (at 1.4 kHz, s.d. 6.8 dB) and 25 dB (at 2.8 kHz, s.d. 9.3 dB).

## Discussion

The appropriate use of the ABR method to achieve auditory measures in harbour seals to low-frequency stimuli has not been previously demonstrated in seals. Wolski et al. (2003) had successfully used this approach at frequencies of 2 kHz and above. To represent a useful threshold estimate, any responses elicited at lower frequencies would have to consist of the same succession of neuronal peaks and troughs (see Jewett and Williston 1971) as those recorded at the higher frequencies and neuronal peaks would also have to appear at increased latencies (Burkard et al. 2007). Moreover, the measured thresholds would have to arrive at a similar level as those achieved with the psychophysical method. The qualitative analysis of the neuronal responses and their latencies measured during stimulation at frequencies down to 1 kHz in this study (see Fig. 2) indicate that these initial requirements were met. The large offset between psychophysical thresholds and ABR thresholds, however, suggests that below 2 kHz the ABR method has its limitations. This may be attributed to a distortion at the level of the basilar membrane, primarily as a spread of activation towards higher frequencies when using relatively high-level stimuli (similar to the upward spread of masking). While this is difficult to assess in the current data, using narrowband stimuli (such as frequency modulated signals) to elicit frequency specific ABRs may allow overcoming this problem.

The auditory measurements of harbour seals presented here revealed aerial hearing thresholds with only relatively small differences between the animals in both test settings (zoo and the wild). These differences may have resulted from the use of different equipment in each setting, differences in background noise levels, different stress levels evoked in the animals in the two settings or reflect population differences. The aerial hearing thresholds are in relatively good agreement with comparable ABR data (after conversion to SPL) published by Wolski et al. (2003). However, in comparison to harbour seal hearing data measured by Reichmuth et al. (2013) in a semi-anechoic chamber using a behavioural technique, differences of more than 80 dB can be found, mainly in the low- and mid-frequency range. This difference can, to some extent, be attributed to non-synchronous firing of neurons along the cochlea (Burkard et al. 2007), a systematic difference between ABR and psychophysical hearing studies (Yuen et al. 2005; Mulsow and Reichmuth 2010). Critical ratios for perception of aerial sounds in the frequency range tested vary in harbour seals from 20 to 25 dB (Turnbull and Terhune 1990; Southall et al. 2000). As all measurements in our study were conducted in the presence of natural masking noise, hearing thresholds at low and mid frequencies are, concurrent to the aforementioned neuronal effects, most likely masked by the level of background noise encountered in both test environments. Ruser et al. (2014) tested several grey seals under comparable laboratory conditions resulting in equally elevated hearing thresholds.

There are other specific aspects which may explain some of the differences found. The volume of the headphone calibration system used is tailored to match the volume of the outer ear of humans, not of harbour seals and could potentially lead to a small offset in the received SPLs. However, as the ABR thresholds cannot be regarded as absolute thresholds, this offset was deemed negligible. Moreover, when comparing the audiometric results between individuals as in this study, this offset in threshold would be consistent for all animals tested. Anaesthesia and a possible change in body temperature during the test procedure have been shown to have an effect on the latency of neuronal responses in humans (Manninen et al. 1985). Reichmuth et al. (2007) compared results achieved in harbour seals and Houser et al. (2007) in northern elephant seals (*Mirounga angustirostris*) for different immobilising drugs and over extended periods of time, but did not find any effect on the amplitude and latency of electrophysiological responses (wave V) measured in these species. Mulsow and Reichmuth (2013) documented differences in response amplitude and latency in association with a reduced body temperature under gas anaesthesia in one of the California sea lions (*Zalophus californianus*) they tested. While these studies are not comprehensive enough to rule out any effect, it was considered being not substantial for the outcome of this study.

While, on repeated visual inspection, all animals appeared to have their external auditory canal open during the hearing test, the duct might have been closed internally under motor control or as a reflex (even when immobilised). This would effectively reduce the sound transmission to the middle and inner ear and lead to a decrease in sensitivity. However, such an effect should lead to a loss in hearing sensitivity over the entire hearing range and not decrease with increasing frequency.

Despite the caveats listed above, this study is the first to compare auditory sensitivity at a selected range of low frequencies of a large number of captive and wild harbour seals. Analysing results achieved from several individuals under comparable environmental conditions, in the field as well as under laboratory conditions, indicates that there is both between-subject and within-subject variability in hearing sensitivity in this species (Lauter and Karzon 1990; Terhune 1991; Kastak and Schusterman 1998; Elberling and Don 2007). This variability could theoretically reflect variations within or differences between animals with regard to their physiological noise floor (biological background noise). Such differences in the neuronal responses could negatively affect the signal-to-noise ratio (SNR) in the recorded neuronal waveforms. The SNR increases if physiological noise is reduced, and this would improve the acuity of determining thresholds. Using a regression approach for the quantitative analysis of the neuronal responses reduced the influence of different levels of biological background noise. The advantage of regression analysis is that thresholds are comparable even if the noise floor is different between subjects as long as the input–output function is linear across the range of data used in the regression. As this condition was met in our analysis, variations in physiological noise floor were unlikely to influence our results.

Progressive hearing loss with increasing old age, presbyacusis, has been reported before in marine mammals (Schusterman et al. 2002; Houser and Finneran 2006). In this context, the elevated hearing thresholds in the two old harbour seals tested in the laboratory setting is not surprising and can likely be attributed to this form of age-related loss in hearing sensitivity. However, it is unusual that the residual hearing sensitivity was found at the high-frequency end of the normal aerial hearing range as presbyacusis normally affects those frequencies first, while hearing sensitivity in the low frequencies remains for longer.

Theoretically, the poor hearing sensitivity in the free-ranging seals from The Wash could stem from exposure to intense underwater sound as found in seismic exploration, underwater explosions, shipping (Richardson et al. 1995), acoustic deterrent devices (Götz and Janik 2013) or offshore pile driving during wind-farm construction (Hastie et al. 2015). However, hearing thresholds in the captive environment were comparable to those in the wild. It is possible that both sample populations had poor hearing and that this is what our data reflect. We think this is unlikely, since captive seals had not been exposed to intensive noise (or ototoxic drugs), and suggest that the elevated thresholds were mainly due to masking and methodological issues. The North Sea has been the subject to substantial acoustic disturbance through construction and oil exploration as well as shipping. The fact that in comparison the hearing thresholds of the wild animals studied here showed no marked difference to that of a captive control group suggests that these seals either have an effective noise avoidance strategy or have not been exposed to substantial noise pollution. Seals have been found to use anthropogenic structures (Russell et al. 2014) and anthropogenic signals that indicate locations of interest (Stansbury et al. 2015). It is likely that they also developed strategies to minimise noise exposure by avoiding high exposure locations (Russell et al. 2016). However, such choices may only be viable if there are alternative, suitable habitats available. Future studies need to investigate the relationships between noise effects, animal avoidance strategies, and habitat availability as well as requirements by the animals, to understand animal tolerance to noise exposure. Such studies should also aim to further develop the ABR method for low-frequency hearing tests and, thereby, make it a more stable method to assess hearing in marine mammals.
